# Z-Scheme CuO_x_/Ag/TiO_2_ Heterojunction as Promising Photoinduced Anticorrosion and Antifouling Integrated Coating in Seawater

**DOI:** 10.3390/molecules28010456

**Published:** 2023-01-03

**Authors:** Xiaomin Guo, Guotao Pan, Lining Fang, Yan Liu, Zebao Rui

**Affiliations:** 1 School of Chemical Engineering and Technology, Guangdong Engineering Technology Research Center for Platform Chemicals from Marine Biomass and Their Functionalization, Sun Yat-sen University, Zhuhai 519082, China; 2 Department of Environmental Science, Hebei University of Environmental Engineering, Qinhuangdao 066102, China

**Keywords:** antibacterial effect, antifouling effect, CuO_x_/Ag/P25, photoelectrochemical cathodic protection, bifunctional coatings

## Abstract

In the marine environment, steel materials usually encounter serious problems with chemical or electrochemical corrosion and fouling by proteins, bacteria, and other marine organisms. In this work, a green bifunctional Z-scheme CuO_x_/Ag/P25 heterostructure coating material was designed to achieve the coordination of corrosion prevention and antifouling by matching the redox potential of the reactive oxygen species and the corrosion potential of 304SS. When CuO_x_/Ag/P25 heterostructure was coupled with the protected metal, the open circuit potential under illumination negatively shifted about 240 mV (vs. Ag/AgCl) and the photoinduced current density reached 16.6 μA cm^−2^. At the same time, more reactive oxygen species were produced by the Z-shape structure, and then the photocatalytic sterilization effect was stronger. Combined with the chemical sterilization of Ag and the oxide of Cu, the bacterial survival rate of CuO_x_/Ag/P25 was low (0.006%) compared with the blank sample. This design provides a strategy for developing green dual-functional coating materials with photoelectrochemical anticorrosion and antifouling properties.

## 1. Introduction

Metal corrosion and fouling in the marine environment are very serious because of the presence of oxygen, Cl^−^ ions, sunlight, and micro-organisms [1,2,3], causing huge economic losses and significant harm to the human living environment. Corrosion generally results from chemical or electrochemical reactions between the metal and oxygen, while the metal surface submerged in seawater or in the humid marine atmosphere is often fouled by marine organisms such as proteins, bacteria, algae, and mollusks, which usually increase the metal weight and accelerate material surface damage [4,5].

Various methods, such as coatings [6], corrosion inhibitors [7], and cathodic protection [8], have been proposed to help reduce metal corrosion in the marine environment. However, these traditional anticorrosion techniques usually suffer from the high cost of resources or additional energy, together with the problem of environmental pollution. Photoelectrochemical cathodic protection (PECCP) technology, which couples a semiconductor material with the protected metal, is a new and green anticorrosion technology [1,3,9]. During the PECCP process, under illumination the semiconductor material generates photoelectrons, which are transferred to the protected metal surface to make the metal potential more negative than its corrosion potential. The typical semiconductor materials, TiO_2_ [2,10,11], ZnO [12], and SrTiO_3_ [9,13], have been evaluated for the PECCP process. Among them, TiO_2_ is one of the most widely used photoelectric anode materials, and it has the advantages of high photoelectrochemical activity, low cost, high stability, and nontoxicity [14,15]. However, TiO_2_ has a low utilization rate of sunlight (<5%), with a relatively wide band gap (~3.2 eV) [16] and a fast recombination rate of photogenerated carriers, which seriously limits its application in the PECCP field. Currently, forming a heterojunction by coupling narrow-band-gap metal oxides with TiO_2_ is an effective way to improve the absorption of visible light and promote the separation of electron/hole pairs [17,18]. Tian et al. [3] deposited Cu_2_O nanoparticles on the surface of TiO_2_ nanotubes to prepare the Cu_2_O/TiO_2_ p-n heterojunction composite photoelectrodes to accelerate the photogenerated carriers’ separation and improve PECCP performance.

Regarding the protection against microbial corrosion, the addition of antibacterial components to the protective paints or coatings has been commonly used to achieve antifouling and antibacterial effects [19]. Although many heavy metals and rare earth elements, such as Cu, Ag, Ce, La, etc., have a strong bactericidal effect [20,21,22], their adverse effects on the environment need to be considered [23]. It is reported that some semiconductors can be also used for photocatalytic sterilization and antifouling, which is correlated with the generation of reactive oxygen species (ROS, such as superoxide radicals •O_2_^−^ and hydroxyl radicals •OH) under irradiation and their inhibition to the growth of bacteria [24]. Wang et al. [24] reported that AgVO_3_/BiO_2−x_ inactivated bacteria in full spectrum, which is due to a large amount of ROS caused by internal structural defects and the formation of heterostructures. Moreover, the integration of chemical sterilization with photocatalytic sterilization has become a key research goal in the antimicrobial field [25,26,27]. Yang et al. [25] designed Cu_2_O/Ag composites with strong and long-term antibacterial activities.

Apparently, both the PECCP performance and photocatalytic antibacterial properties of semiconductors are related to the light absorption, photoinduced charges separation, and the conduction/valence-band positions. In this sense, we herein design a bifunctional CuO_x_/Ag/TiO_2_ Z-type heterojunction, which is anticipated as coating material for metal anticorrosion/antifouling in the marine environment on the basis of the following considerations. (i) Both CuO_x_ and TiO_2_ hold higher conduction-band positions against the potential of stainless steel (e.g., 304SS) and make the transfer of photoinduced electrons to the metal for PECCP possible. (ii) The combination of Ag nanoparticles (NP) with a local surface plasmon resonance (LSPR) effect and small-band-gap CuO_x_ can improve the visible light absorption of TiO_2_ [28,29]. (iii) The unique Z-type CuO_x_/Ag/TiO_2_ heterojunction provides an effective charge transfer path for realizing the efficient separation of electron-hole pairs without sacrificing redox ability [30]. (iv) The bacteria, viruses, and other micro-organisms can be inhibited or chemically killed by Ag and CuO_x_ and by active ROS groups produced by photoinduced electrons and holes from the heterojunction [25,26,27,31,32]. As expected, the as-designed CuO_x_/Ag/TiO_2_ not only well demonstrates photocathodic protection performance for 304SS in the simulated seawater but also possesses chemical and photocatalytic synergistic bactericidal activities. Such a green bifunctional coating material shows great promise in metal anticorrosion and antifouling applications in the marine environment.

## 2. Results and Discussion

### 2.1. Structures, Compositions, and Morphologies

**Figure 1:**
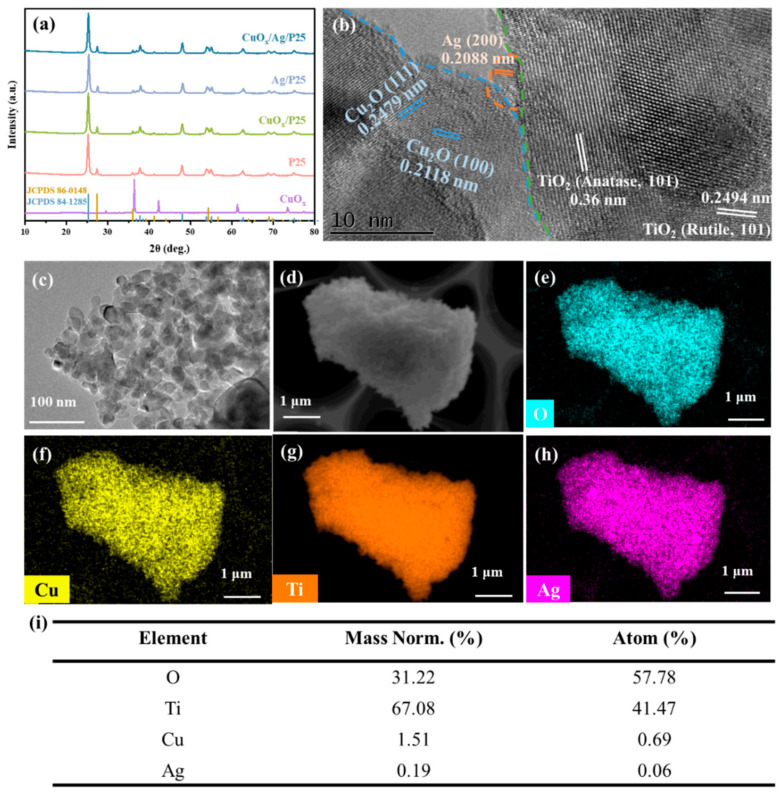
(**a**) XRD patterns of P25, CuO_x_, CuO_x_/P25, Ag/P25, and CuO_x_/Ag/P25; (**b**) HRTEM and (**c**) TEM images of CuO_x_/Ag/P25; (**d**–**h**) EDS element mapping images of CuO_x_/Ag/P25; (**i**) list of element content of CuO_x_/Ag/P25.

XRD measurements were employed to study the crystalline structures of samples. As shown in Figure 1a, the diffraction peaks for P25 could be assigned to anatase and rutile TiO_2_ (JCPDS 84-1285 and JCPDS 86-0148). The diffraction peak intensity at 2θ = 25.3 ° was strong, and the peak shape was symmetrical, indicating that the crystallinity of the material is very good. The reference material CuO_x_ presented characteristic diffraction peaks at 2θ = 36.4 and 42.3 °, corresponding to the (111) and (200) crystal planes of the Cu_2_O phase of cubic hematite (JCPDS 05-0667). Compared with P25 and CuO_x_, the diffraction peaks for CuO_x_/Ag/P25 remained unchanged, indicating that CuO_x_ and Ag did not affect the crystal structure of P25, which may have been due to the small load, the small particle size, and the low crystallinity of CuO_x_ and Ag [3,33]. The morphologies and nanostructures of CuO_x_/Ag/P25 were analyzed by TEM and HRTEM. As shown in Figure 1b, the lattice spacings of 2.5 and 3.6 Å corresponded to the (101) crystal plane of anatase TiO_2_ and the (101) crystal plane of rutile TiO_2_, respectively. The lattice spacings of 2.1 and 2.4 Å were ascribed to the (100) and (111) crystal planes of Cu_2_O [3,16,34]. The (200) crystal plane of Ag with a lattice spacing of about 2.08 Å was found at the interface between the CuO_x_ phase and the P25 phase [35]. Figure 1c shows that CuO_x_/Ag/P25 was mainly composed of irregular particles with a diameter of 20~25 nm. The EDS element distribution mapping in Figure 1d exhibited that the Ti, O, Ag, and Cu elements were evenly distributed in CuO_x_/Ag/P25. The corresponding element content is listed in Figure 1i.

**Figure 2:**
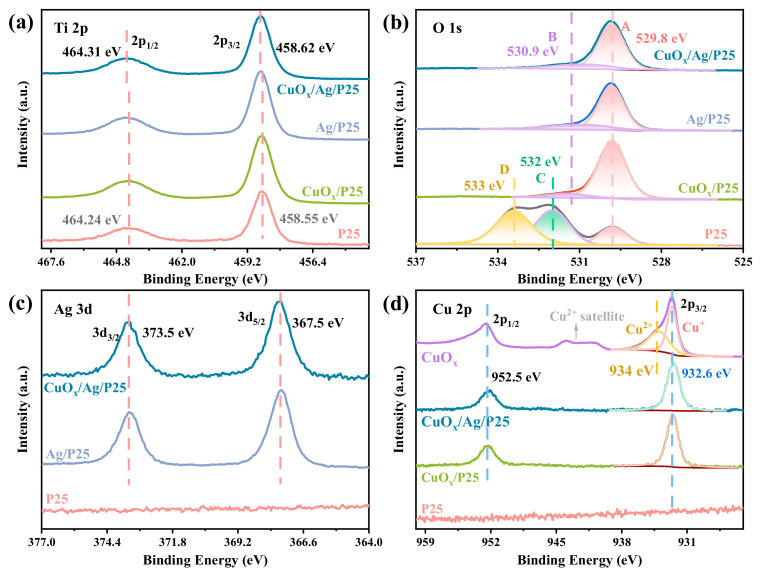
XPS spectra of the samples: (**a**) Ti 2p, (**b**) O 1s, (**c**) Ag 3d, and (**d**) Cu 2p.

The atomic valence states and energy-band structures of CuO_x_/P25, Ag/P25, and CuO_x_/Ag/P25 were further studied by XPS. The Ti 2p spectra for P25 in Figure 2a showed a pair of spin-orbital doublets at ~464.2 and ~458.5 eV, corresponding to Ti 2p_1/2_ and Ti 2p_3/2_ of Ti^4+^, respectively [3,28,36]. Compared with P25, the peaks for CuO_x_/Ag/P25 showed a shift to a higher binding energy, which was possibly due to the strong interaction between CuO_x_, Ag, and P25 in the heterojunction structure of CuO_x_/Ag/P25 [37]. The O1s spectra of the samples shown in Figure 2b can be divided into four types of peaks in total. Peak A, around 529.8 eV, belonged to the oxide peak, which was related to the lattice oxygen in TiO_2_. The slightly shifted peak B, at ~530.9 eV, also belonged to the metal (Ag and Cu) oxide peak, which was attributed to the O atom near the oxygen vacancy [38]. Peak C, around ~532 eV, belonged to the -OH group, chemically adsorbed on the material surface [39], and peak D, around ~533 eV, belonged to adsorbed H_2_O molecules on the surface [40]. Figure 2c shows that the Ag 3d spectra of Ag/P25 and CuO_x_/Ag/P25 can be deconvoluted into two peaks, Ag 3d_3/2_ (373.5 eV) and Ag 3d_5/2_ (367.5 eV), indicating the existence of Ag metal in the material [30]. In the Cu 2p spectra for CuO_x_ (Figure 2d), the peaks with binding energies of 934 and 932.6 eV can belong to the Cu^2+^ [41] and Cu^+^ [42] species, respectively. In addition, the satellite peaks corresponding to Cu^2+^ species can be seen between 940 and 945 eV. This indicates that Cu^+^ and Cu^2+^ coexisted in CuO_x_. For CuO_x_/P25 and CuO_x_/Ag/P25, only peaks at 932.6 eV corresponding to the binding state of Cu^+^ species were observed, which demonstrates that the strong interaction between the carrier (P25) and the load (CuO_x_ and Ag) made the Cu species stable.

### 2.2. Photoelectric Characterization

**Figure 3:**
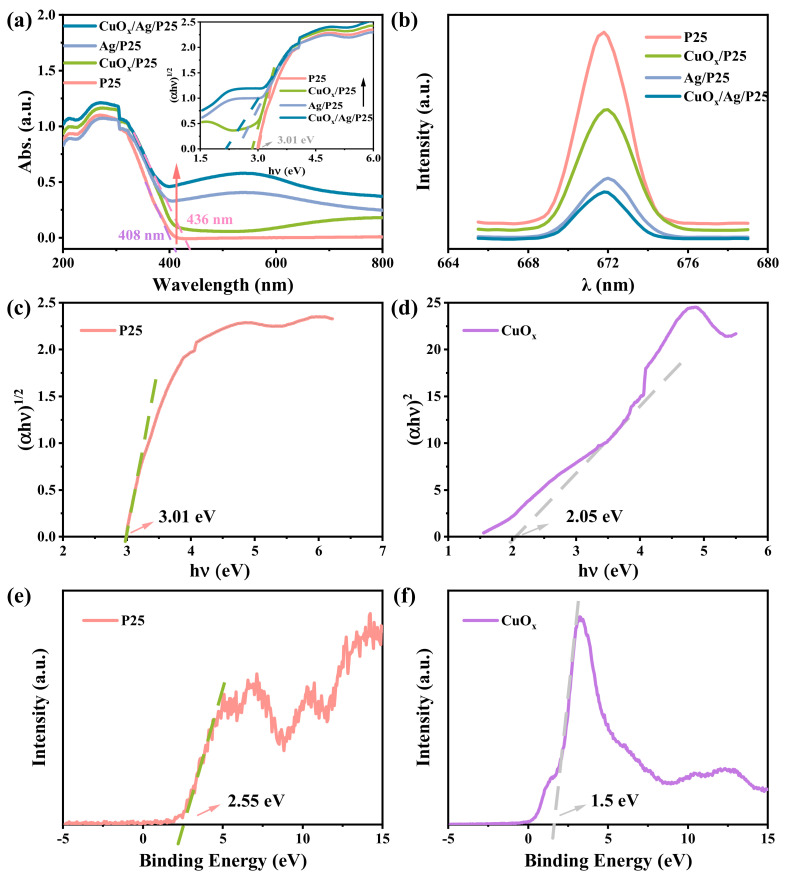
(**a**) UV−vis DRS, dependence of (αhν)^2^ on the photon energy (the inserts) and (**b**) photoluminescence spectra (λ_ex_ = 550 nm) of P25, CuO_x_/P25, Ag/P25, and CuO_x_/Ag/P25 at room temperature. Dependence of (αhν)^2^ on the photon energy for (**c**) P25 and (**d**) CuO_x_. XPS valence-band spectra of (**e**) P25 and (**f**) CuO_x_.

UV-visible diffuse reflectance spectra (or UV-vis DRS) were measured to investigate the light absorption of samples. Figure 3a showed a typical TiO_2_ light-absorption range. The light-absorption range of as-prepared CuO_x_/Ag/P25 was extended to the visible region with an edge of about 436 nm after loading CuO_x_ and/or Ag. At the same time, it can be observed that Ag/P25 and CuO_x_/Ag/P25 had strong and wide absorption peaks near 550 nm, which were related to the LSPR effect of Ag NPs [43,44]. Figure 3a shows the band-gap results of the materials. CuO_x_/Ag/P25 holds the narrowest band gap (2.17 eV) compared with P25 (3.01 eV), CuO_x_/P25 (2.87 eV) and Ag/P25 (2.49 eV). The photoluminescence (PL) spectra of the catalysts at an excitation wavelength of 550 nm at room temperature were displayed in Figure 3b. The fluorescence peak intensity for CuO_x_/Ag/P25 was lower than that of other compound materials, indicating the lowest recombination rate of e^−^/h^+^ pairs. The fluorescence intensity order was CuO_x_/Ag/P25 < Ag/P25 < CuO_x_/P25 < P25, which suggested that having Ag NP as the electron bridge promoted the separation and emigration of photogenerated electron-hole pairs [30,45]. By plotting the Kubelka–Munk function against the photon energy, the band0gap (*E*g) values of P25 and CuO_x_ were estimated to be 3.01 and 2.05 eV, respectively (Figure 3c,d). Moreover, the *E*g values of the composites, such as Ag/P25, CuO_x_/P25, and CuO_x_/Ag/P25, became narrower after loading CuO_x_ and Ag, which confirmed the broadened light-response range. According to the XPS valence-band (or XPS-VB) spectra in Figure 3e,f, the corresponding *E*_VB,XPS_ of P25 and CuO_x_ were 2.55 and 1.5 eV, respectively. The VB positions of P25 and CuO_x_ were calculated as 2.31 eV and 1.26 eV, respectively, by using the following equation: *E*_VB,NHE_ = φ + *E*_VB,XPS_ − 4.44, where φ is the work function of the instrument (4.2 eV [46]).

**Figure 4:**
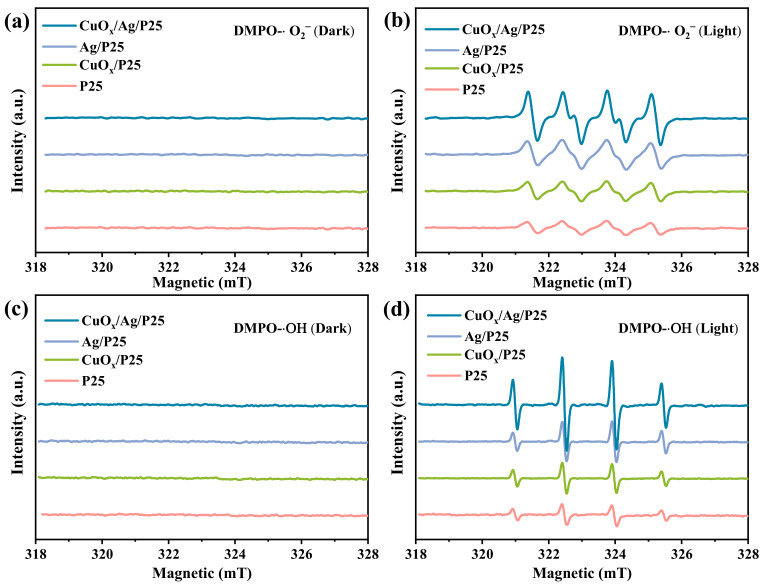
EPR spin-trapping spectra of DMPO−•O_2_^−^ and DMPO−•OH adducts (**a**,**c**) in the dark and (**b**,**d**) under illumination, respectively, for P25, CuO_x_/P25, Ag/P25, and CuO_x_/Ag/P25. The light used was full-spectrum light.

The electron paramagnetic resonance (EPR) spectra of free radical adducts trapped by DMPO in the dark and under irradiation at room temperature for the catalysts are shown in Figure 4. The EPR signals for DMPO−•OH and DMPO−•O_2_^−^ were almost negligible on P25, CuO_x_/P25, Ag/P25, and CuO_x_/Ag/P25 in darkness. Both DMPO−•OH and DMPO−•O_2_^−^ signals with four characteristic peaks with intensity ratios of 1:2:2:1 and 1:1:1:1 [47], respectively, can be observed in the suspension under simulated solar irradiation for 12 min. EPR signals of DMPO−•OH and DMPO−O_2_•^−^ were different in intensity. The intensity order of DMPO−•O_2_^−^ signals under irradiation was CuO_x_/Ag/P25 > Ag/P25 > CuO_x_/P25 > P25; the intensity order of DMPO−•OH signals under irradiation was CuO_x_/Ag/P25 > Ag/P25 ≈ CuO_x_/P25 > P25. CuO_x_/Ag/P25 showed the highest intensity in the characteristic peaks of DMPO−•OH and DMPO−•O_2_^−^ when compared with those materials.

### 2.3. Photoelectrochemical Cathodic Protection Performance Evaluation

**Figure 5:**
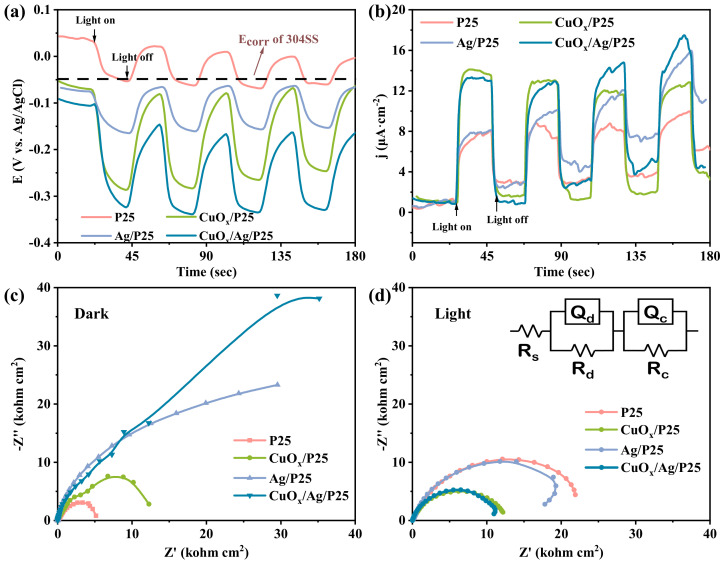
(**a**) The photoinduced OCP−time curves and (**b**) i−t curves of P25, CuO_x_/P25, Ag/P25, and CuO_x_/Ag/P25 coupled with 304SS. Nyquist plots measured (**c**) in the dark and (**d**) under illumination. Insert of (**d**) is the corresponding equivalent circuit.

In general, photogenerated cathodic protection works by supplying electrons to the protected metal through an external photosensitive semiconductor, causing the metal potential to be more negative than the original corrosion potential under irradiation. Thus, the photoinduced OCP value is an important parameter to evaluate the PECCP performance of photoanode materials. Figure 5a shows the photogenerated OCP-time curves of samples. The photoelectrodes that were coupled with 304SS were used as working electrodes. P25 showed the minimum negative potential shift of 90 mV under light on/off cycles, which was a little lower than the self-corrosion potential of 304SS (−50 mV), indicating that P25 hardly achieved PECCP toward 304SS. The negative shift order of OCP values for samples was CuO_x_/Ag/P25 > CuO_x_/P25 > Ag/P25 > P25. Compared with CuO_x_/P25 and Ag/P25, CuO_x_/Ag/P25 displayed a significantly more negative potential shift of 240 mV under illumination, indicating that the introduction of CuO_x_ and Ag improved the separation efficiency of electron-hole pairs, and more electrons were transferred to the coupled 304SS to provide photoelectrochemical cathodic protection. Figure 5b shows the photoelectric response of P25, CuO_x_/P25, Ag/P25, and CuO_x_/Ag/P25 coupled with 304SS in a 3.65 wt.% NaCl solution with an applied bias potential of 0 V (*E_ref_*). P25, CuO_x_/P25, Ag/P25, and CuO_x_/Ag/P25 showed a positive current density change when the light was turned on, showing the characteristics of an n-type semiconductor [48]. It indicates that photoinduced electrons could be transferred from the semiconductor materials to the coupled 304SS. Compared with P25, the photocurrent densities of CuO_x_/P25 and CuO_x_/Ag/P25 greatly increased at the first light on/off switching. At the same time, using Ag particles as a conducting medium accelerated the separation of electron-hole pairs [3,30]. Thus, CuO_x_/Ag/P25 had a photocurrent density of 16.6 μA cm^2^, which was ~1.8 times greater than that of P25, indicating that it had the best PECCP performance. The migration ability of photogenerated carriers could be characterized by electrochemical impedance spectroscopy (EIS).

Figure 5c,d show EIS results measured in the dark and under illumination, respectively. In darkness, the impedance arc radius of CuO_x_/Ag/P25 was larger than that of the other materials, which indicates that the thin film electrode could be used as a coating material to protect 304SS from corrosion [18]. Under illumination, the smaller the impedance arc radius of the impedance spectrum is, the faster the photogenerated carriers migrate, resulting in better PECCP properties [49]. Figure 5d shows that CuO_x_/Ag/P25 had the smallest arc radius, demonstrating the best carriers transfer efficiency and thus the greatest photochemical cathodic protection effect, which was consistent with the results of OCP response and the photoinduced current. In the corresponding equivalent circuit model, *R*_s_, *R*_d_, and *R*_c_ represented the solution resistance, the depletion layer resistance, and the charge transfer resistance [50], respectively. The fitting results in Table 1 showed that CuO_x_/Ag/P25 had a higher *R*_d_ value than P25, which may have been due to the bending of the Fermi level caused by the formation of a Z-type heterostructure. Moreover, CuO_x_/Ag/P25 had an *R*_c_ value of 1.23 Ω, much smaller than P25 (2.47 × 10^4^), CuO_x_/P25 (1.22 × 10^4^), and Ag/P25 (300.5), respectively. Therefore, it could be understandable that CuO_x_/Ag/P25 had the best PECCP performance for 304 SS in a 3.65 wt.% NaCl solution.

### 2.4. Antibacterial Performance Evaluation

**Figure 6:**
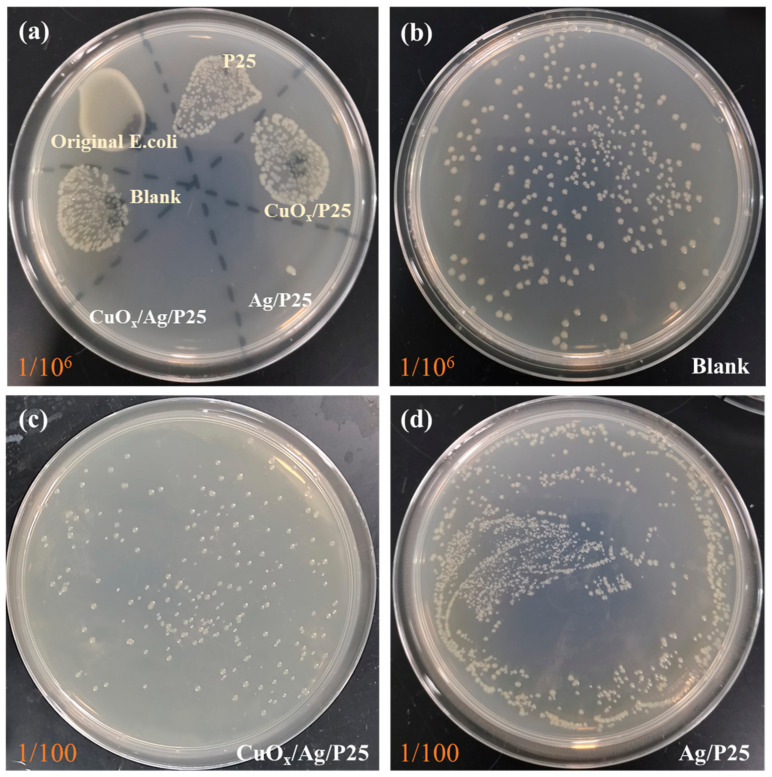
Optical photographs of agar plates coated with different catalysts and *E. coli* incubated solution with various dilutions: (**a**) comparison of the samples at 1 × 10^6^–times dilution, (**b**) blank sample at 1 × 10^6^–times dilution, (**c**) CuO_x_/Ag/P25 at 100-times dilution, and (**d)** Ag/P25 at 100-times dilution.

**Figure 7:**
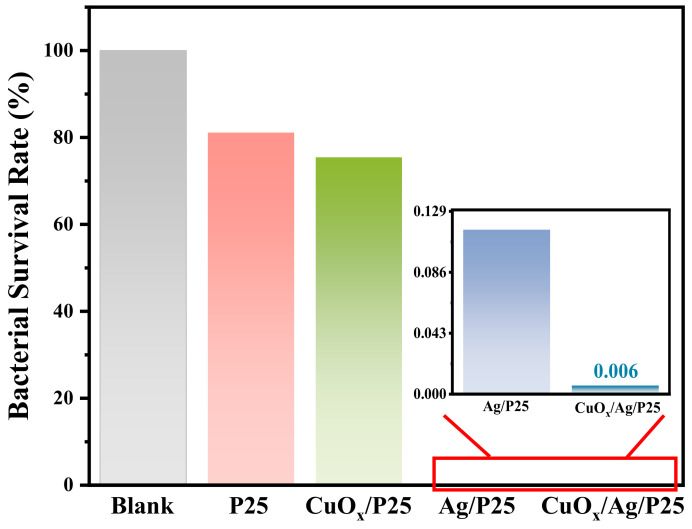
Comparison diagram of the bacterial survival rate in the media with different catalysts.

The antibacterial activities of the materials were evaluated by using the CFU method, i.e., an *E. coli* colony–counting method. The optical pictures of *E. coli* colonies on nutrient agar plates in Figure 6a exhibited that CuO_x_/Ag/P25 and Ag/P25 coatings had fewer *E. coli* colonies than the other samples did, indicating better antibacterial activities under 24 h of illumination. Taking the blank sample without active catalysts as a counterpart in Figure 6b, it can be calculated that the bacterial survival rate of P25 and CuO_x_/P25 in a 1 × 10^6^–times diluted bacterial solution were about 81.1% and 75.4%, respectively. Moreover, as shown in Figure 6c,d and Figure 7, the bacterial survival rates of Ag/P25 and CuO_x_/Ag/P25 in a 100-times diluted solution were as low as 0.116% and 0.006%, respectively. It is concluded that CuO_x_/Ag/P25 can effectively inhibit the growth of *E. coli*.

## 3. Discussion

**Figure 8:**
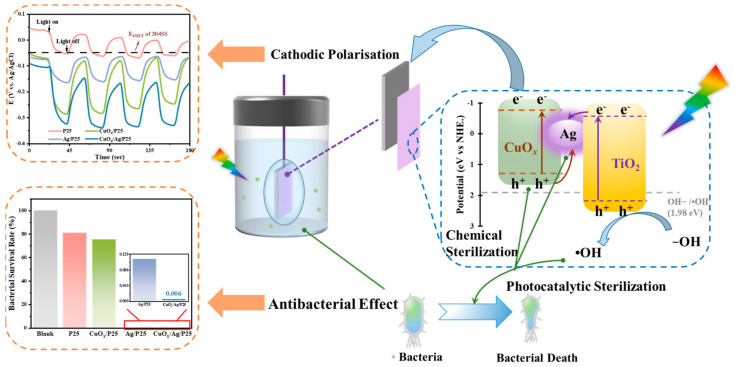
Schematic diagram of the antibacterial and anticorrosion dual mechanism for Cu_x_O/Ag/P25.

The energy-band positions of CuO_x_ (−0.79 eV/1.26 eV) and P25 (−0.7 eV/2.31 eV) determined in this work (Figure 3c–f) and the reported Fermi level of Ag NP (~−0.22 eV) [44] are listed in Figure 8, indicating that a Z-scheme or type-II heterojunction structure was formed in CuO_x_/Ag/P25 [28,30]. Moreover, because the VB potential of CuO_x_ is not positive enough for the production of •OH from H_2_O oxidation, the stronger DMPO-•OH signal of CuO_x_/Ag/P25, compared with that of Ag/P25 (Figure 4d), indicates that CuO_x_/Ag/P25 is inclined to form a Z-scheme system in which the photoinduced electrons in Ag/P25 transfer and combine with the holes on the valance band (VB) of CuO_x_, leaving the photoinduced holes at the VB of P25 for the production of •OH and the photoinduced electrons at the conduction band (CB) of CuO_x_ for the photocathode protection and production of superoxide ions (O_2_•^−^). Additionally, the incorporation of Ag NP with the LSPR effect and CuO_x_ with a narrow band gap greatly improved the visible light absorption (Figure 3a). All these together promoted the production of photoinduced charges and the ROS of •O_2_^−^ and •OH radicals (Figure 4) under illumination. On one hand, the efficient production and transfer of the photoinduced charges in the unique Z-scheme heterojunction of CuO_x_/Ag/P25 makes effective the transfer of photogenerated electrons from the CB of CuO_x_ to the coupled 304SS, achieving enough of a negative shift of the 304SS potential and photoelectrochemical cathodic polarization protection toward the metal (Figure 5a). On the other hand, their strong ROS (superoxide radicals •O_2_^−^ and hydroxyl radicals •OH) production ability upon irradiation and their ability to inhibit the growth of bacteria, viruses, and other micro-organisms provide the antifouling ability of the CuO_x_/Ag/P25 coating [26,27]. Moreover, both CuO_x_ and Ag NPs, especially the Cu_2_O species stabilized by the photoinduced electrons through the unique Z-scheme transfer path (Figure 2d), effectively maintain chemical disinfection properties [25,31,32]. The superior antibacterial performance of CuO_x_/Ag/P25 and their application potential as an antifouling coating are demonstrated in Figure 6 and Figure 7.

## 4. Experimental Section

### 4.1. Synthesis of CuO_x_/Ag/TiO_2_

First, noble metal Ag was loaded on TiO_2_ (P25) by using the wet chemical reduction method. Typically, 0.5 g of P25 was ultrasonically dispersed in 30 mL of deionized water. Then 5 mL of AgNO_3_ aqueous solution with a concentration of 1 mg mL^−1^ was added to the above suspension under vigorous stirring. After fully mixing, an appropriate amount of NaBH_4_ aqueous solution was added dropwise to reduce Ag^+^. The molar ratio of NaBH_4_ to AgNO_3_ was 4:1. Under continuously stirring for 4 h, the resulting powder was obtained by centrifugation and washing with deionized water and absolute ethanol, separately, several times, followed by drying in an oven at 80 °C to obtain the Ag/P25 sample with a theoretical Ag loading of 1.0 wt.%.

Then, CuO_x_ was loaded on P25 or Ag/P25 by using the deposition-precipitation method. Next, 0.3 g of P25 or Ag/P25 and 0.0074 g of CuSO_4_·5H_2_O were added into 60 mL of 0.2 mol mL^−1^ NaOH aqueous solution under magnetic stirring for 30 min, followed by the addition of 9.0 mL of 0.1 mol L^−1^ ascorbic acid aqueous solution, drop by drop. After reaction for 1.5 h, the product was centrifuged and washed with deionized water and absolute ethanol, separately, until the solution was neutral. At last, 1.0 wt.% CuO_x_/P25 or 1.0 wt.% CuO_x_/Ag/P25 was obtained after drying the sample overnight in the flowing N_2_ at 80 °C.

### 4.2. Preparation of Coatings

The as-prepared sample was mixed with nafion/ethanol solution under ultrasonic treatment for the preparation of the coating material [51]. Typically, 1 mL of absolute ethanol was mixed with 100 μL of 5% nafion to obtain the solvent. Next, 0.5 mg of CuO_x_/Ag/P25 (or other sample) was then ultrasonic dispersed in the above 200 μL solvent. The as-obtained ultrasonic slurry was coated on FTO glass substrates with a coating area of 1 cm × 1 cm. Finally, the photoanode coating was obtained after drying in an oven at 40 °C for 3 h.

### 4.3. Characterizations

The crystal structures of the prepared powders were analyzed by an X-ray powder diffractometer (Rigaku Ultima IV) equipped with a Cu Kα radiation source. The diffraction patterns in the range of 10 to 80° were recorded at a scan speed of 10° min^−1^. The morphologies and element distribution of the samples were observed by high-resolution thermal field emission scanning electron microscope (FESEM, Gemini500). The crystal interface structures of the samples were analyzed by high-resolution transmission electron microscope (HRTEM, FEI Tecnai G2 F30) with an accelerating voltage of 300 kV. The element composition and valence state of the catalysts were analyzed by X-ray photoelectron spectroscopy/ESCA (XPS, Thermo Fisher Scientific, Nexsa). A monochromatic Al Kα X-ray source (h*υ* = 1486.6 eV) under a vacuum degree of ~2 × 10^−9^ mbar was used. The surface pollution C1s (284.8 eV) was used as the standard for energy correction. The UV-vis diffuse reflectance spectroscopy (UV-vis DRS) response of catalysts in the wavelength range from 200 to 800 nm was analyzed by integrating a sphere UV-vis spectrophotometer (UV2600) with BaSO_4_ as a reference. Photoluminescence (PL) spectra were characterized by using Edinburgh FS5 fluorescence spectrometer with an excitation wavelength of 550 nm. Electron paramagnetic resonance (EPR) spectrometer (JES X320) was used to obtain signals of photogenerated radicals spin trapped by 5,5-dimethyl-1-pyrroline-N-oxide (DMPO), e.g., DMPO-•OH and DMPO-O_2_•^−^, in the dark and under illumination (190–900 nm) at room temperature. The free radical signals were collected after 12 min.

### 4.4. Photoelectrochemical Measurements

The electrochemical measurements were performed at room temperature using Gamry electrochemical workstation (Gamry Interface1010E). All measurements were carried out after the open circuit potential (OCP) value was stable. A 300 W Xenon lamp (CEL-HXF300) was used as the light source with an optical power density of 200 mW cm^−2^. In a three-electrode system, Pt plate was used as a counter electrode, Ag/AgCl electrode (saturated KCl) was used as a reference electrode, and the photoanode (catalyst coatings on FTO glass substrates, i.e., P25, Ag/P25, CuO_x_/P25, or CuO_x_/Ag/P25) was used as a working electrode. In the PECCP performance measurements, such as those from the OCP and photocurrent density-time (i-t) tests, the photoanode coupled with 304SS was used as a working electrode. Additionally, a 3.65 wt.% NaCl solution was used as an electrolyte solution to simulate the marine environment. Mott–Schottky (M–S) curves were measured in the potential scope of −1.5 to 0.2 V at a frequency of 1000 Hz. Electrochemical impedance spectroscopy (EIS) was performed in the AC voltage of 10 mV and the frequency range from 10^5^ to 10^−2^ Hz with or without light illumination.

### 4.5. Antibacterial Performance Evaluation

The antibacterial activities of the materials were evaluated by using the CFU method (a plate-counting method) using *Escherichia coli* (*E. coli*) in a beef-extract-peptone (BEP) medium at 37 °C for 24 h. The concentration of the bacterial suspension was adjusted to 3 × 10^7^~3 × 10^8^ CFU mL^−1^. The catalysts were dispersed by deionized water to a concentration of 2 mg mL^−1^. The mixture of 1 mL of catalyst, 1 mL of BEF, and 100 μL of *E. coli* suspensions was cultured on a shaking table (ZQLY-180GN, 150 rpm) under simulated solar irradiation at 37 °C for 24 h (16000LX). At the end of the incubation period, the culture medium was sampled to determine the viable counts of planktonic bacteria. The viable bacteria in the sampled suspension were counted by using a 10-times gradient dilution method. Specifically, 100 μL of a diluted sample was transferred to an LB agar plate, which was cultured in a bacterial incubator (MJX-150) at 37 °C for 24 h. Finally, the colonies were counted, and the antibacterial activities of the catalysts were compared according to the bacterial survival rate α, as calculated by


$$\alpha =\frac{\mathrm{Number}\;\mathrm{of}\;\mathrm{viable}\;\mathrm{bacteria}\;\mathrm{in}\;\mathrm{the}\;\mathrm{control}\;\mathrm{sample}\;\times \;\mathrm{Dilution}\;\mathrm{ratio}}{\mathrm{Number}\;\mathrm{of}\;\mathrm{viable}\;\mathrm{bacteria}\;\mathrm{in}\;\mathrm{the}\;\mathrm{blank}\;\times \;\mathrm{Dilution}\;\mathrm{ratio}}\times \mathrm{100\%}$$

## 5. Conclusions

In summary, we have successfully prepared CuO_x_/Ag/P25 coatings on FTO glass substrates, which displayed excellent photocathodic protection performance and antifouling activities under simulated solar illumination in a 3.65 wt.% NaCl solution. The photoelectrochemical measurements and characterizations revealed that a Z-type CuO_x_/Ag/P25 heterostructure provided a photogenerated carrier transfer channel, which facilitated carrier separation and resulted in a more negative OCP shift of 240 mV. Meanwhile, the Z-type heterojunction of coatings had high-conduction-band and deep-valance-band potentials, which generated more reactive oxygen species of •O_2_^−^ and •OH radicals, thus effectively killing *E. coli* bacteria, with a low survival rate of 0.006%.

## Figures and Tables

**Table 1:** EIS fitting results of photoanode films.

	*R*_s_ (Ω)	*R*_d_ (Ω)	*R*_c_ (Ω)
P25	9.24	31.68	2.47 × 10^4^
CuO_x_/P25	10.12	239.7	1.22 × 10^4^
Ag/P25	11.50	2.10 × 10^4^	300.5
CuO_x_/Ag/P25	8.51	1.21 × 10^4^	1.23

## Data Availability

The data presented in this study are available on request from the corresponding author.

## Sample Availability

Samples of the compounds are not available from the authors.
